# Genomic scale analysis of NK cells impact on response to IFN-α

**DOI:** 10.1186/2051-1426-1-S1-P46

**Published:** 2013-11-07

**Authors:** Maria L Ascierto, Harvey Alter, Federica Bozzano, Antonio Picciotto, Simona Marenco, Francesco Marras, Cathy Schechterly, Davide Bedognetti, Valeria De Giorgi, Michele Sommariva, Paolo A Ascierto, Lorenzo Moretta, Ena Wang, Francesco M Marincola, Andrea De Maria

**Affiliations:** 1 National Institutes of Health, Bethesda, MD, USA; 2 University of Genoa, Genoa, Italy; 3 Instituto Nazionale Tumori, Naples, Italy; 4 Sidra Medical and Research Center, Doha, Qatar

## 

Recently, several works have shown that both the innate and adaptive immunity contribute to different responsiveness to cancer therapy. Similar portraits have been described in autoimmunity diseases and viral infections. Based on these observations we here explored in hepatitis C (HCV) infected patients the role of NK cells in therapy response. For the last decade, the standard treatment for HCV infection has been limited to IFN-α+ ribavirin (IR) combination therapy. Recently immunogenetic aspects regarding inhibitory receptors of NK cells (KIRs): HLA and IL-28B have been partly associated to IR response. However, the ability to couple the screening of these markers with other easily measured biomarkers may make the prediction more sensitive and specific resulting very useful in the clinical setting. In the current study we conducted high throughput screening of NK cells derived from healthy individuals and chronically infected HCV-1 patients prospectively collected before undergoing IR treatment and we identified that the treatment outcome of HCV patients is associated with the expression pattern of molecules involved in post-transcriptional modifications of RNA/protein trafficking. With the development of a highly stringent prediction model we identified gene signatures whose expression was able to predict with 100% accuracy the outcome of treatment. Among the predictive genes, snoRNAs genes were playing a major role suggesting an unexpected relevance of the non-coding RNAs (ncRNAs) in clinical outcome of HCV patients. These data indicate that the relevance of the non-coding genome is not limited to microRNA expression and function; instead also other not coding RNAs (i.e.:snoRNAs) represent key elements of cellular homeostasis and immune responses. Moreover, our results support in humans the existence of RNA-based gene- expression regulatory system carried by introns and other non-coding genomic regions which, in HCV infection, is associated with diverging treatment response. Altogether our results showed that NK cells evaluation to HCV patients provide comprehensive explanation of useful determinants of clinical response. Thus, the usage of the NK molecular signatures could identify the proportion of chronically infected patients who would most benefit from IR treatment and it could also be further applied for the screening of predictive parameters of HCV-associated conditions (i.e. hepatocarcinoma).

